# Efficient spin filtering through Fe_4_GeTe_2_-based van der Waals heterostructures†

**DOI:** 10.1039/d4na00639a

**Published:** 2024-10-09

**Authors:** Masoumeh Davoudiniya, Biplab Sanyal

**Affiliations:** a Department of Physics and Astronomy, Uppsala University Sweden Biplab.Sanyal@physics.uu.se

## Abstract

Utilizing *ab initio* simulations, we study the spin-dependent electronic transport characteristics within Fe_4_GeTe_2_-based van der Waals heterostructures. The electronic density of states for both free-standing and device-configured Fe_4_GeTe_2_ (F4GT) confirms its ferromagnetic metallic nature and reveals a weak interface interaction between F4GT and PtTe_2_ electrodes, enabling efficient spin filtering. The ballistic transport through a double-layer F4GT with a ferromagnetic configuration sandwiched between two PtTe_2_ electrodes is predicted to exhibit an impressive spin polarization of 97% with spin-up electrons exhibiting higher transmission probability than spin-down electrons. Moreover, we investigate the spin transport properties of Fe_4_GeTe_2_/GaTe/Fe_4_GeTe_2_ van der Waals heterostructures sandwiched between PtTe_2_ electrodes to explore their potential as magnetic tunnel junctions in spintronic devices. The inclusion of monolayer GaTe as a 2D semiconducting spacer between F4GT layers results in a tunnel magnetoresistance of 487% at a low bias and decreases with increasing bias voltage. Overall, our findings underscore the potential of F4GT/GaTe/F4GT heterostructures in advancing spintronic devices based on van der Waals materials.

## Abstract 2

*Ab initio* simulations reveal that Fe_4_GeTe_2_/GaTe heterostructures with PtTe_2_ electrodes exhibit high spin polarization and 487% tunnel magnetoresistance, demonstrating strong spin filtering and potential for spintronic applications.

## Graphical abstract

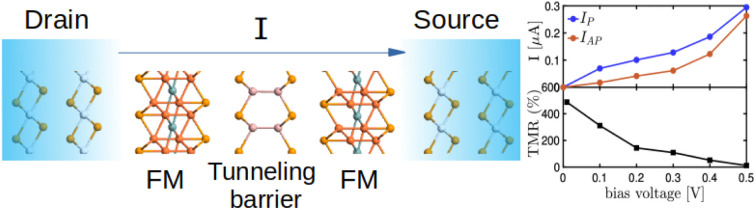

## 1 Introduction

Quantum transport through two-dimensional (2D) magnetic structures has emerged as a fascinating field of research with promising implications for spintronics.^1–7^ Spintronic aims to harness both the charge and spin degrees of freedom to enable the development of novel electronic devices with enhanced functionalities. Moreover, 2D magnetic structures, such as atomically thin ferromagnetic films and magnetic heterostructures, possess distinct spin-dependent properties that enable efficient control and manipulation of electron spins. Understanding spin transport in these systems is crucial for the development of spin-based electronic devices and spintronic circuits. Historically, most magnetic tunnel junctions (MTJs) were constructed using perovskite-oxide materials. However, these conventional MTJs have limitations, notably a large resistance-area product, which restricts their practicality in device applications.^8^ In contrast, van der Waals (vdW) materials have shown promise in overcoming the challenges associated with traditional magnetic thin films. They lead to significantly high tunnel magnetoresistance (TMR) values, as evidenced by numerous experimental studies.^9–14^ The vdW heterostructures, composed of atomically thin layers stacked on top of each other, have emerged as promising platforms to explore and exploit such quantum transport phenomena.^2,15–23^ The weak vdW forces facilitate the formation of a clean and atomically sharp interface between layers, enabling efficient transfer of spin-polarized electrons between the magnetic materials.

Recent discoveries of Fe_*n*_GeTe_2_ (*n* = 3, 4, 5) (FGT), a class of 2D itinerant ferromagnets with a Curie temperature approaching room temperature, provide exciting prospects for 2D spintronic advancements.^24–27^ A comparative study of FGT family has been done in ref. 28 with the aid of *ab initio* calculations. FGT exhibits a notable advantage stemming from its metallic nature, which facilitates the manipulation of both electronic spin and charge. Furthermore, FGT has been suggested as a rare-earth-free material with strong magnetism and electronic correlation.^29^ Recent experimental reports have confirmed that the tunneling resistance behavior in hBN sandwiched between two Fe_3_GeTe_2_(F3GT) layers follows established patterns, exhibiting minimum (maximum) resistance when the magnetizations of the electrodes are parallel (antiparallel). Remarkably, a significant magnetoresistance of 160% is observed at low temperatures, indicating a spin polarization of 0.66 in F3GT.^18^ Moreover, the formation of ohmic contacts in F3GT/MoS_2_ interfaces is confirmed by linear current–voltage curves, indicating a conducting layer rather than a tunneling one. This is a positive result as ohmic contacts enable efficient charge transport with minimal resistance, whereas tunneling contacts can obstruct current flow and reduce device performance. It has been also observed that magnetoresistance of F3GT/MoS_2_/F3GT heterostructures reaches 3.1% at 10 K, which is approximately 8 times larger than conventional spin valves with MoS_2_ and conventional ferromagnetic electrodes.^30^ While F3GT has been extensively explored, the focus on Fe_4_GeTe_2_ (F4GT) has been relatively limited, despite its higher Curie temperature, making it a promising avenue for further research.^31^ Investigating F4GT can provide valuable insights into its exceptional electronic, magnetic, and structural properties. By understanding the distinctive characteristics of F4GT, one can unlock its potential for technological applications such as spintronics, magnetic storage, and other advanced electronic devices. FGT-based heterostructures are highly promising for spintronic devices due to their strong spin-filtering and spin polarization, making them ideal for MTJs and spin valves, which are critical components in advanced non-volatile memory technologies like magnetoresistive random access memory (MRAM).^5,11,32,33^ The tunable magnetic properties and spin–orbit coupling in the FGT family also enhance their use in spin–orbit torque devices, enabling efficient, high-speed magnetic switching for logic and memory applications.^34^ These materials are valuable in quantum computing as spin filters, facilitating selective spin transmission essential for spin-based quantum gates and complex computing systems. Moreover, FGT's high sensitivity to magnetic fields makes it useful for magnetic sensors and detectors, boosting performance in automotive, aerospace, and other industrial technologies.^35^

Here, the nonequilibrium Green's function (NEGF) formalism and *ab initio* simulations were utilized to analyze the transport characteristics of vdW heterostructures consisting of F4GT. This approach is a robust method for studying ballistic transmission across ultrathin magnetic films.^2,36–46^ The focus of this study is also on investigating the electronic and magnetic properties of these heterostructures. Specifically, we investigated spin-dependent ballistic transport in both mono- and bi-layer F4GT structures that were sandwiched between PtTe_2_ electrodes. To assess the tunneling magnetoresistance behavior, we analyzed the spin-dependent electronic transport across F4GT/GaTe/F4GT junctions, connected to PtTe_2_ electrodes, serving as vdW MTJs.

## 2 Methodology

We employed the QuantumATK software package^47^ to investigate quantum transport properties. The calculations involved the combination of density functional theory (DFT) and the NEGF formalism. The DFT calculations were carried out using the generalized gradient approximation (GGA) to describe the exchange–correlation functional. We obtained the electronic band structure and density of states (DOS) of F4GT to gain insights into its electronic properties. Realistic device structures, including the scattering region and leads, were constructed to simulate the transport properties. The NEGF formalism was employed to calculate the transmission spectra and current–voltage characteristics of the F4GT-based devices under external biases. To accurately account for vdW interactions, we applied the DFT-D3 method with Becke–Jonson damping.^48,49^ Structural relaxations were performed using the linear combination of atomic orbitals (LCAO) basis set with PseudoDojo for pseudopotentials. A Monkhorst–Pack grid of 14 × 14 × 1 has been used and a cutoff density of 140 hartree was chosen to ensure convergence. Moreover, a force tolerance of 10^−3^ eV Å^−1^ was used for relaxations. Convergence was achieved when the total energy difference between consecutive steps was below 10^−4^ eV. The source and drain electrodes were set to a length of 10.402 Å, and the same LCAO settings were applied for the quantum transport simulations. For the gate-all-around (GAA) structure, the Poisson solver utilized the Dirichlet boundary condition in all directions. We utilized a Monkhorst–Pack grid with dimensions of 16 × 16 × 1 to assess the transmission and current. To maintain reasonable simulation times, the parallel conjugate gradient method was employed. To calculate the magnetocrystalline anisotropy energy (MAE), we included spin–orbit coupling (SOC) using a full *k*-point grid, and the Brillouin zone integration was performed using a 55× 55 × 1 *Γ*-centered Monkhorst–Pack grid. Moreover, previous studies^28^ have demonstrated that GGA + U calculations are incompatible with experimental results for FGT materials. This incompatibility extends to parameters such as unit cell dimensions, magnetic moments, magnetic anisotropy energy, and transition temperature. Consequently, utilizing static electron correlation is not suitable for accurately characterizing a metallic magnet like FGT. Therefore, we have neglected the Hubbard U correction in our calculations.

The spin-dependent transmission coefficient was determined using Green's functions, as expressed by the following equation^50^1
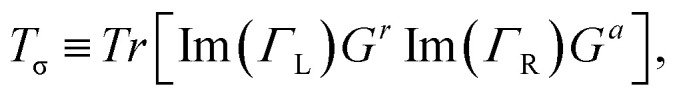
The subscript *σ* ≡ ↑,↓ represents the spin index. The term 
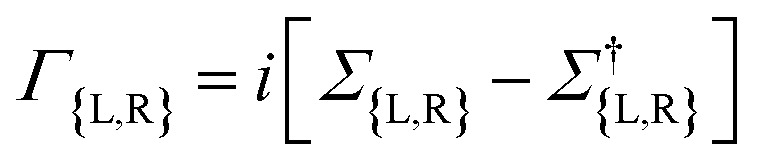
 is the line width function and *Σ*_L(R)_ in the equation corresponds to the retarded self-energy of the left (right) electrode, representing the coupling between the central region and the semi-infinite leads. This term accounts for the interaction and exchange of electrons between the central region and the electrodes. The *G*^*r*(*a*)^ term refers to the retarded (advanced) Green's function matrices, which describe the propagation of electrons through the system in the spin and orbital spaces.

To find the transmission eigenstates, we utilize a linear combination of Bloch states, *∑*_*n*_*e*_α*n*_*ψ*_*n*_, with coefficients *e*_α*n*_ that diagonalize the transmission matrix. This transmission matrix is mathematically defined as2
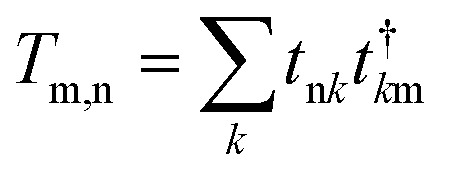
Here, *t*_n*k*_ represents the transmission amplitude, indicating how likely an electron in Bloch state *ψ*_n_ on the left electrode will have a transition to Bloch state *ψ*_*k*_ on the right electrode. The transmission coefficient can be computed by taking the trace of *T*_m,n_.

To calculate the spin-dependent tunneling current, the Landauer–Büttiker formula was employed,^51^3
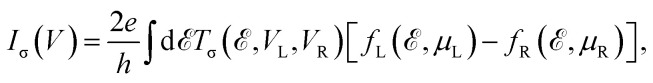


The tunneling current is determined by the electrochemical potentials *μ*_L_(*μ*_R_), Fermi distribution functions *f*_L_(*f*_R_), and bias voltages *V*_L_(*V*_R_) applied to the left (right) lead at room temperature. The transmission coefficient 
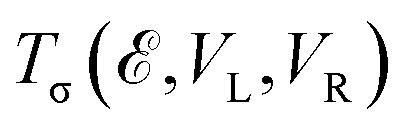
 is energy-dependent and varies with the bias voltages and energy of the system. In the calculations of current for the device mentioned in this paper, we used a voltage-dependent transmission function. This means the transmission function is evaluated at each bias voltage, taking into account the changes in electronic structure and transmission properties under finite bias. This approach ensures that the calculated current accurately reflects the influence of the applied voltage, rather than relying on the zero-bias transmission function. We performed the current calculations at room temperature (300 K). For the transmission and electric current calculations, the cross-sectional area of the device is defined as the transverse unit cell area, which is perpendicular to the direction of electron transport. We report the total transmission and electric current for each spin channel, indicating that the current density is multiplied by this cross-sectional area.

## 3 Results and discussion

### 3.1 Characterization of PtTe_2_/F4GT/PtTe_2_ heterostructure

**Fig. 1:**
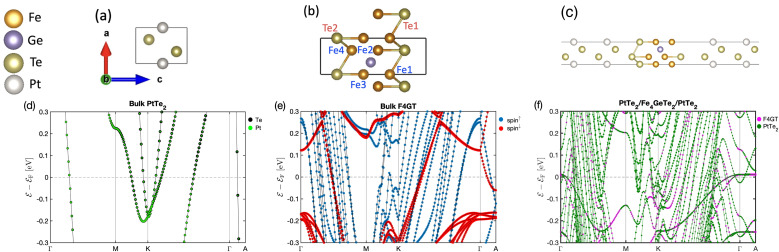
The atomic structures of (a) PtTe_2_, (b) F4GT, and (c) F4GT sandwiched between two PtTe_2_ electrodes. Panels (d–f) showcase the corresponding electronic band structures without considering spin–orbit coupling: (d) atom-projected electronic band structure for bulk PtTe_2_, (e) spin-projected electronic band structure for bulk F4GT, and (f) layer-projected electronic band structure for monolayer F4GT sandwiched between two PtTe_2_ electrodes.

The unit cell of PtTe_2_ is depicted in Fig. 1a. It possesses a layered crystal structure within the trigonal space group *P*3̄*m*1.^52^ The structure comprises Pt atoms situated between two layers of Te atoms. The stacking of these layers repeats in a hexagonal pattern along the *c*-axis, generating a three-dimensional structure. The interlayer interactions are governed by weak vdW forces. The lattice constants obtained using the GGA functional are *a* = *b* = 4.01 Å and *c* = 5.201 Å. Fig. 1b indicates the layered crystal structure of F4GT, which shares the same space group (*P*3̄*m*1) as PtTe_2_.^53^ Each layer consists of four Fe atoms surrounded by Ge and Te atoms. The Ge and Te atoms form a distorted hexagonal lattice, with the Fe atoms occupying the centers of distorted octahedra formed by the coordination with Ge and Te. Magnetic moment of Fe_1_ (Fe_4_) and Fe_2_ (Fe_3_) have been calculated to be 2.72 *μ*_B_ and 1.71 *μ*_B_, respectively, which is in good agreement with ref. 54. The calculated lattice constant of F4GT is found to be *a* = *b* = 3.968 Å, which is remarkably close to the lattice constant of PtTe_2_. This similarity in lattice constants suggests a strong structural resemblance between F4GT and PtTe_2_, indicating potential similarities in their crystal structures and bonding arrangements. We initiate our investigation by examining a two-probe system consisting of a single-layer F4GT situated between two PtTe_2_ electrodes (Fig. 1c). In this setup, the F4GT layer is subjected to a tensile strain of 0.7%. The distance between the Te atoms on the surface of F4GT and the surface layer of PtTe_2_ is approximately 2.88 Å. This distance is larger than the interlayer distances in bulk PtTe_2_, which is around 2.33 Å, and smaller than the interlayer distances in bulk F4GT, which is approximately 3.28 Å. Moreover, in the device configuration, the magnetic moment of Fe_1_ and Fe_4_ remains unchanged at 2.72 *μ*_B_. However, the magnetic moment of Fe_2_ and Fe_3_ slightly increases to 1.81 *μ*_B_.

Fig. 1d displays the atom-projected band structure plots of the monolayer PtTe_2_. Additionally, Fig. 1e presents the spin-polarized bands of F4GT, while Fig. 1f shows bands projected onto both the total F4GT layer and the PtTe_2_ layer of the PtTe_2_/F4GT/PtTe_2_ system. The band structure analysis reveals that both monolayer PtTe_2_ and F4GT exhibit metallic behavior. In the case of F4GT, the states around the Fermi level are primarily dominated by the spin-up channel, accompanied by a smaller contribution from the spin-down channel. As observed in Fig. 1f of the weight-projected band structure plot, numerous bands intersect and cross the Fermi level. These crossing points indicate the presence of potential conductance channels within the device configuration. The majority of states near the Fermi level originate from Te and Pt atoms from PtTe_2_ layers. This difference can be attributed to the larger number of Te and Pt atoms present in the electrodes of the device, leading to a higher contribution from these elements to the electronic states near the Fermi level. Furthermore, upon closer examination near the Fermi level at the *M* and *K* points (see Fig. 1f), it is evident that the F4GT bands in the device configuration exhibit minimal changes compared to the isolated F4GT (see Fig. 1e). This observation suggests a weak vdW interaction at the interface between F4GT and the electrodes. The relatively unchanged Fe bands indicate that the electronic structure of F4GT is preserved within the device, implying that the interface interaction does not significantly affect the Fe electronic states.

**Fig. 2:**
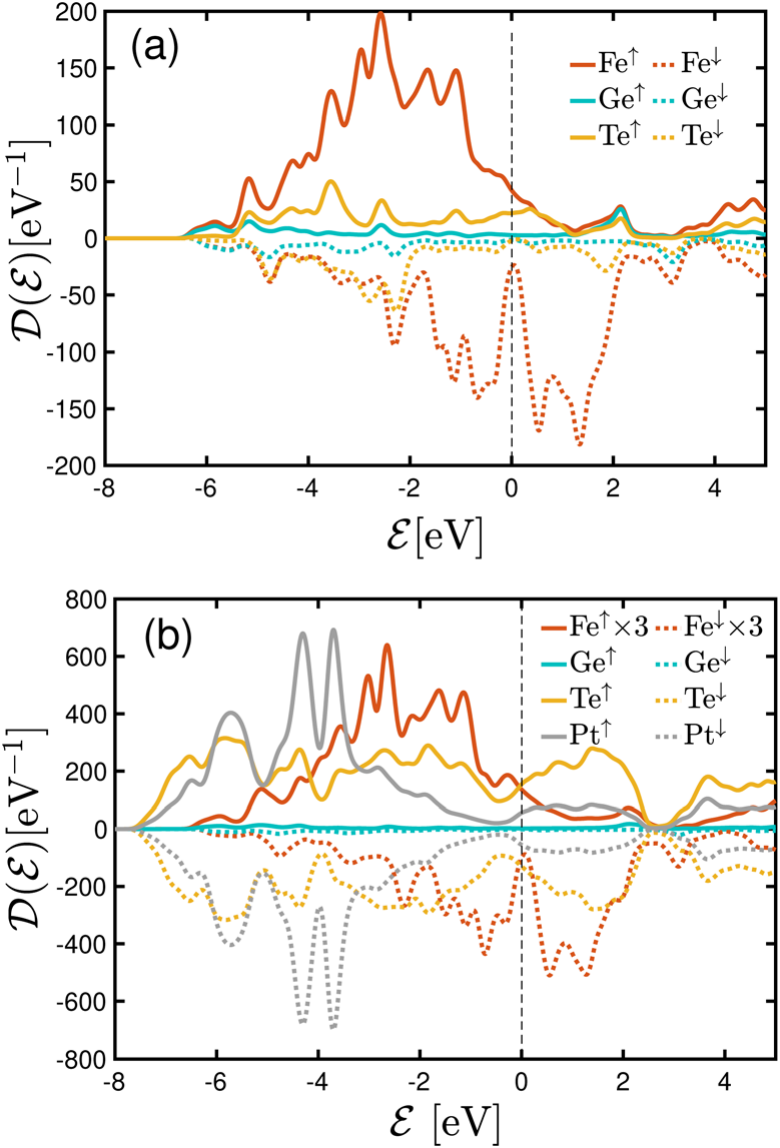
DOS plots for (a) the freestanding monolayer F4GT and (b) the F4GT sandwiched between two layers of PtTe_2_.

The spin-polarized DOS calculations were performed to investigate the spin-dependent electronic properties of the monolayer F4GT (a) and the F4GT sandwiched between two PtTe_2_ electrodes (b), as shown in Fig. 2. Panel (a) reveals a significant electron density at the Fermi level 
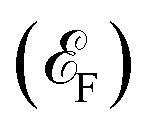
 with an exchange splitting, indicating the ferromagnetic nature of F4GT. This finding is consistent with previous *ab initio* calculations.^55^ The states near the Fermi level are primarily dominated by the Fe atoms in F4GT, contributing to the majority spin channel. The orbital-decomposed d-band DOS for the 3d orbitals of Fe atoms in a freestanding monolayer of F4GT, shown in Fig. S1,† reveals significant contributions at the Fermi level. The d_*xz*_ and d_*z*^2^_ orbitals exhibit pronounced spin-split peaks near the Fermi level, with the spin-up states being more prominent than the spin-down states. The d_*xy*_ orbitals also contribute, though less significantly. In panel (b), the DOS plot showcases the impact of the PtTe_2_ layers on the electronic states of the F4GT sandwiched structure. It is observed that the redistribution and shifting of the electronic states in the Fe atoms are relatively unchanged, indicating a weak interaction between the F4GT and PtTe_2_ layers. This suggests that the electronic properties of F4GT remain largely preserved within the device configuration. Furthermore, the DOS values from panel (b) highlight the spin filtering mechanism of the device configuration. The electronic states at 
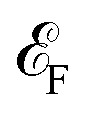
 in the majority spin channel are more abundant compared to the minority spin channel. This indicates the potential for spin-polarized currents when a bias voltage is applied to the device.

To assess the impact of SOC on the transport properties of the device, we have calculated the noncollinear transmission coefficient for the device composed of F4GT sandwiched between two PtTe_2_ electrodes, as demonstrated in Fig. S3.† Our findings indicate that SOC has a negligible effect on the transmission coefficient of the device. This can be primarily attributed to the use of non-magnetic electrodes in our calculations. The magnetic properties influencing transport are derived from the Fe atoms in the F4GT layer. For the purpose of focusing on transport phenomena, we intentionally excluded SOC from our consideration in the model.

### 3.2 Spin filtering

**Fig. 3:**
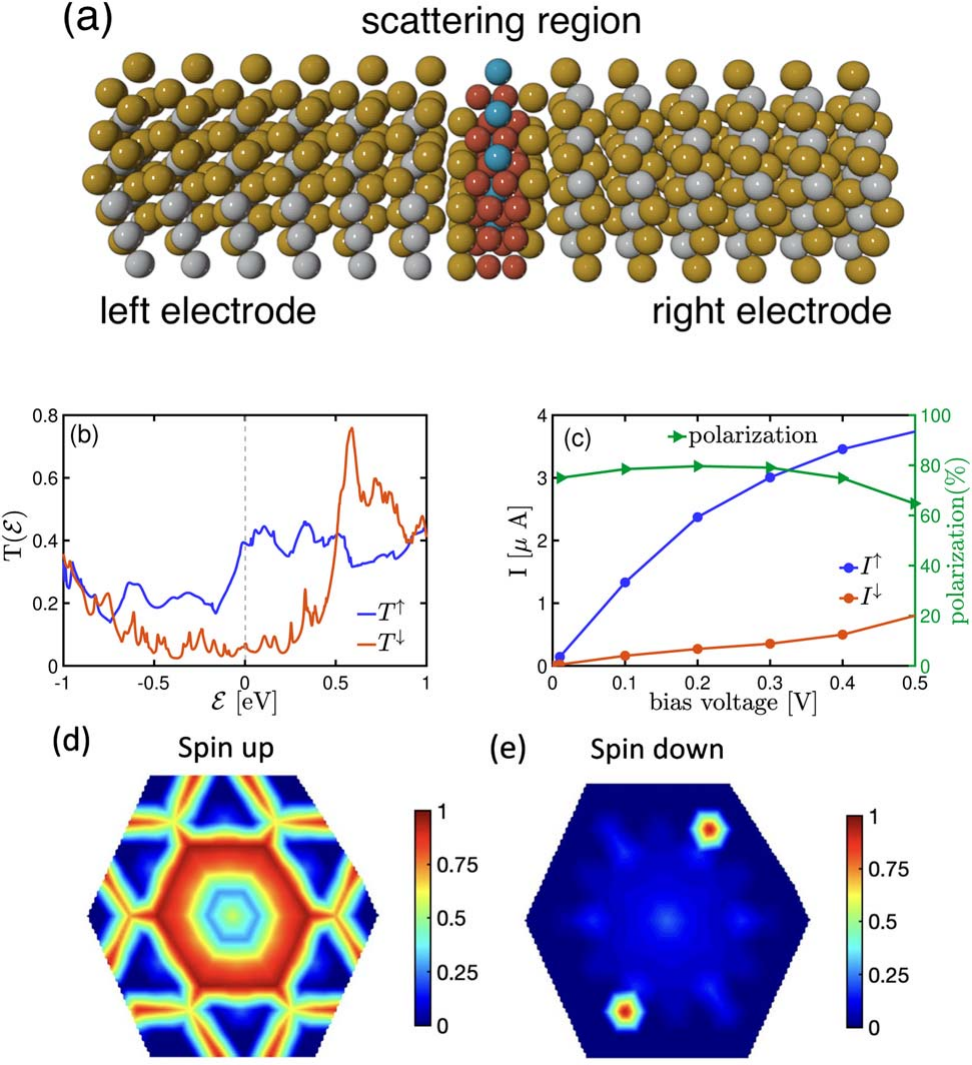
(a) Schematic representation of a two-probe model for NEGF calculations, depicting a monolayer F4GT sandwiched between two PtTe_2_ electrodes. (b) The transmission spectrum of the system under zero bias voltage. (c) Variation of spin polarization, spin-up current (*I*↑) and spin-down current (*I*↓) with bias voltage. The *k*_‖_-resolved transmission probability for (d) spin-up and (e) spin-down electrons at the Fermi energy in the absence of bias voltage.

Fig. 3 presents the transmission spectrum of a monolayer F4GT sandwiched between two PtTe_2_ electrodes (panel a) under zero bias voltage. The results show ballistic transport near the Fermi level with distinct spin polarization (Fig. 3b). In the vicinity of 
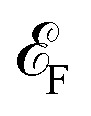
, the transmission coefficient for the spin-up channel is higher, while the spin-down channel exhibits a broad transmission peak reaching its maximum value of 0.75 at 
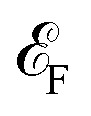
 +0.6 eV. In contrast, the transmission coefficient for the spin-up channel remains comparatively lower at this energy level. This behavior is also supported by the DOS plot in Fig. 2, where a peak for the spin-down channel is observed at the same energy level (
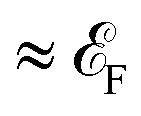
 +0.6 eV). This peak results in a higher contribution of the spin-down channel to the transport properties of the system. The difference in the transmission behavior between the two spin channels strongly indicates the presence of spin-polarized transport properties in the F4GT-based device configuration. This can be seen in Fig. 3c, which exhibits a non-zero spin-polarized current for both spin channels. The *I*–*V* curve shows that the spin-up channel has a higher value of current, indicating a preferential flow of electrons with specific spin orientations. Similar spin filtering behavior in F3GT has been reported in conjunction with Cu electrodes.^4^

Additionally, we have conducted calculations to determine the spin polarization of the current, which is defined as *P* = |*I*↑ − *I*↓|/(*I*↑ + *I*↓). Since we require a finite bias voltage for polarization calculations, we have included polarization plots for all cases starting from 0.01 V throughout the paper. In Fig. 3c, the current spin polarization exhibits an impressive value of 80%, which remains consistently high in the voltage range of 0.1–0.4 V, comparable to the polarization observed at very low bias voltages. However, as the bias voltage is further increased to 0.5 V, the polarization gradually decreases and reaches 64%. This indicates that single-layer F4GT serves as an effective material for achieving substantial spin polarization, particularly at lower bias voltages. The significant transport polarization observed in F4GT agrees well with experimental results. A very recent study employing spin-resolved Andreev reflection spectroscopy on F4GT revealed an exceptionally high transport spin polarization, surpassing 50%.^56^

The *k*_‖_-resolved transmission probability offers a comprehensive understanding of how electrons with different spin orientations propagate through the material at the Fermi level. Thus, we performed calculations to determine the momentum-dependent transmission for Fig. 3d spin-up and Fig. 3e spin-down electrons at the Fermi energy under zero bias voltage. As expected, the transmission probability for spin-up electrons surpasses that of spin-down electrons, indicating a significant degree of spin polarization in the material. Furthermore, we found that the transmission in both the spin-up and spin-down channels does not change with the reversal of *k*_‖_ due to the two-fold rotational symmetry of the system. This is consistent with the structural symmetry of the system.

**Fig. 4:**
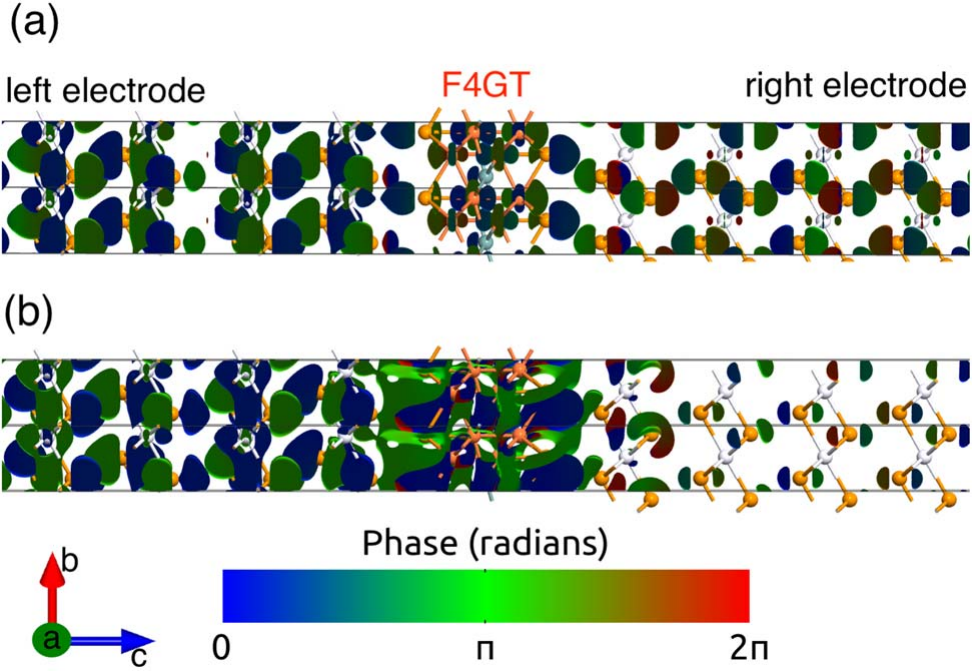
The isosurface plots of transmission eigenstates for a monolayer F4GT placed between two PtTe_2_ electrodes under zero bias voltage at the Fermi level for (a) the spin-up and (b) the spin-down channel. For both channels, the isosurface values are fixed at 0.17 Å^−3^ eV^−1^.

Quantum transmission eigenstates, which characterize electron propagation within a device, are depicted *via* isosurface plots in Fig. 4. The figure shows a monolayer F4GT placed between two PtTe_2_ electrodes for both (a) spin-up and (b) spin-down channels at the Fermi energy level under zero bias. A quantum transmission eigenstate can be thought of as a combination of two distinct electron states. One of these states represents electrons moving from the left electrode to the right electrode, while the other describes electrons going from the right electrode to the left electrode. Their relative phase depends on their proximity to the respective electrodes. This phase difference results in an interference-like pattern in the isosurface plot, particularly in the PtTe_2_ layers on the left and right sides of F4GT, far from the scattering region. A π phase shift is observed for the transmission eigenstates localized on the left electrode in the spin-down channel (panel b) compared to the spin-up channel (panel a). Nevertheless, for the PtTe_2_ layers on the right side of F4GT, the phase of eigenstates is the same for both channels. In Fig. 4a, the transmission eigenstate in the scattering region exhibits a pattern characterized by d_*z*^2^_ orbitals on Fe atoms for the spin-up channel. Interestingly, we observe that in the right electrode region, the transmission eigenstates pertaining to the spin-up channel (panel a) exhibit greater dominance when compared to those of the spin-down channel (panel b).

**Fig. 5:**
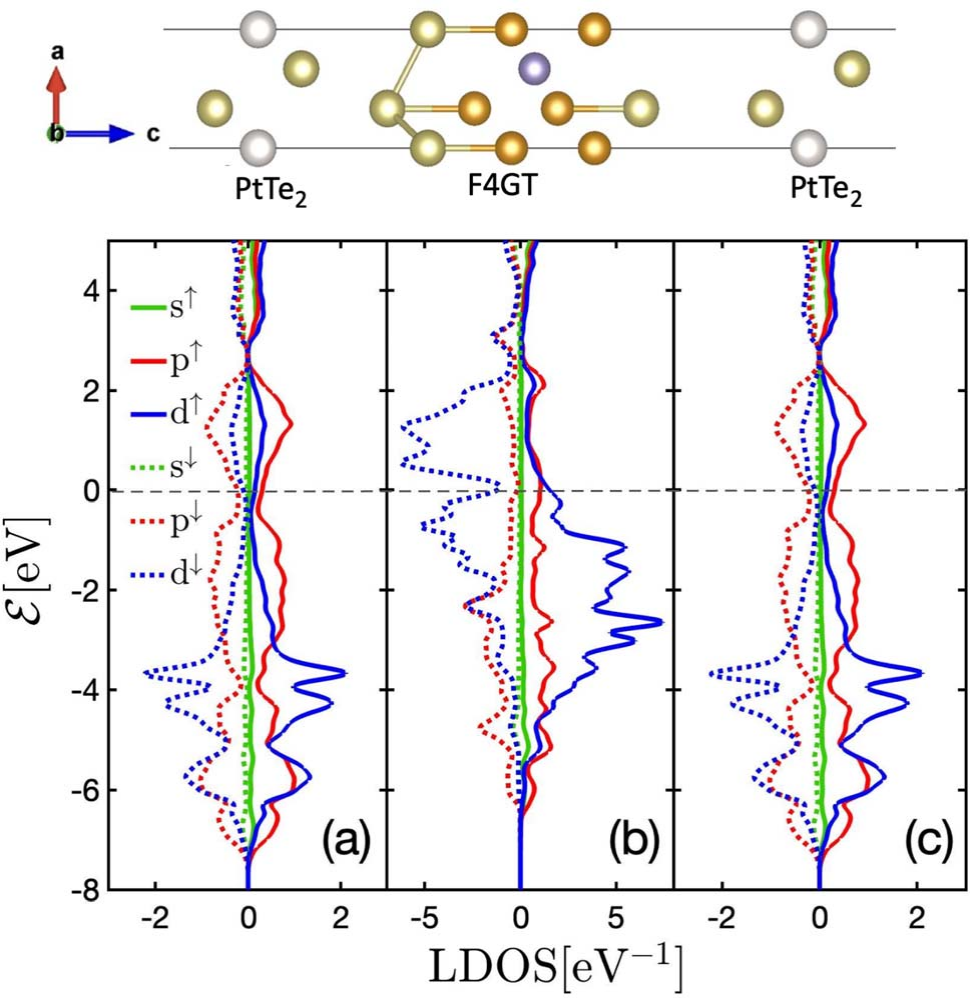
Orbital-projected local density of states for (a) the left electrode's PtTe_2_ layer interfacing with F4GT, (b) F4GT itself, and (c) the right electrode's PtTe_2_ layer interfacing with F4GT.

A comprehensive examination of the fundamental mechanisms contributing to this spin-filtering phenomenon reveals that the dominant transmission eigenstates in the spin-up channel establish a robust transmission channel in the heterojunction. This enables the efficient movement of electrons from the left electrode to the right electrode. Conversely, the spin-down channel experiences a relative scarcity of transmission eigenstates (Fig. 4b), resulting in a restricted transmission of electrons to the right side. The observed spin-filtering effect originates from the channel-selective transmission behavior, where the spin-up channel displays a more pronounced transmission, allowing a larger number of electrons to traverse from the left to the right side. The orbital-projected local density of states is presented in Fig. 5 for distinct orbitals, focusing on (a) the left electrode's PtTe_2_ layer interfacing with F4GT, (b) F4GT itself, and (c) the right electrode's PtTe_2_ layer interfacing with F4GT. The figure highlights a substantial contribution from both p and d orbitals, resulting in discernible d–p hybridization states near the Fermi level.

**Fig. 6:**
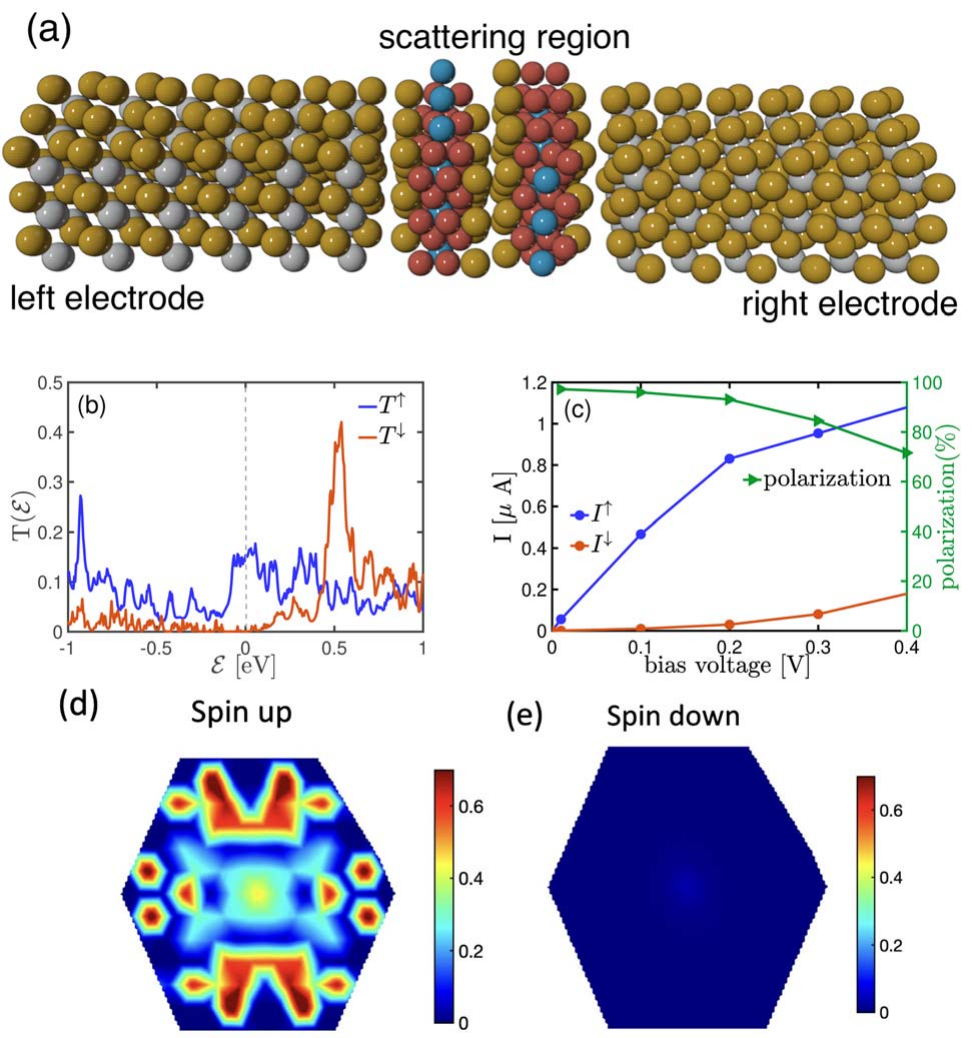
(a) Schematic illustration of the PtTe_2_/bilayer F4GT/PtTe_2_ heterostructure. (b) Spin-dependent transmission coefficient at zero bias in the ferromagnetic (FM) configuration. (c) *I*–*V* characteristics and spin polarization of the heterostructure in the FM configuration. (d) Zero-bias *k*_‖_-resolved transmission probability at the Fermi energy for spin-up electrons. (e) Zero-bias *k*_‖_-resolved transmission probability at the Fermi energy for spin-down electrons.

Furthermore, we have calculated the spin-polarized electronic structure and transport properties for the bilayer F4GT placed between two PtTe_2_ electrodes. The AB-stacking for two F4GT layers is selected due to its higher stability in terms of energy compared to the AA-stacking configuration. The ground state energy for AA-stacked bilayer F4GT is found to be −92.21 eV, whereas the ground state energy for AB-stacked bilayer F4GT is −92.26 eV. The two-probe model for the NEGF calculations of this bilayer system is illustrated in Fig. 6a. Fig. 6b displays the spin-resolved energy-dependent transmission function of the system at zero voltage, with the energy measured relative to the Fermi level and the bilayer F4GT in a ferromagnetic (FM) configuration. Similar to the monolayer system, the zero-bias ballistic transport near the Fermi level exhibits spin polarization. However, in the bilayer system, there is a broad transmission peak between approximately 
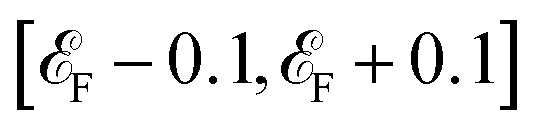
 eV, with its maximum at 
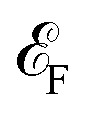
 for the spin-up channel. In contrast, the transmission coefficient in the spin-down channel is suppressed. This behavior results in a spin filtering effect, allowing only one spin type to flow through the constriction at this particular energy range. The *I*–*V* calculation (Fig. 6c) reveals that the difference in spin-up and spin-down currents is more pronounced in the bilayer system compared to the single-layer F4GT, leading to higher polarization of the current. This value of spin polarization surpasses the reported spin polarization values for a device comprising a bilayer of F3GT placed between Cu electrodes. The highest spin polarization value for the Cu/F3GT/Cu heterostructure with a ferromagnetic configuration is documented as 85% at a very low bias voltage.^4^ The significantly enhanced spin polarization in our FM system indicates its potential for efficient spin transport in spintronic devices.

To gain further insight into the transport properties of the systems, we analyzed the distribution of transmission coefficients at the Fermi level under zero bias voltage, as depicted in Fig. 6d and e. In comparison to the spin-down channel shown in panel (e), the 
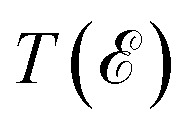
 for the spin-up channel in panel (d) exhibits higher values and is distributed across most of the first Brillouin zone region. However, for the spin-down channel, the transmission contours are primarily concentrated around the *Γ* point. This indicates that the spin-up electrons have a significantly higher probability of transmission, while the transmission of the spin-down electrons is largely suppressed.

### 3.3 Magnetic tunnel junction

The study of MTJs is important in spintronics, as they are key components in spin-based devices.^57^ The structure of an MTJ consists of ferromagnetic layers separated by a tunneling barrier between them. The MTJs can come in different sizes, use low energy, and could potentially last without wearing out. These properties make the MTJ highly valuable for various applications like MRAM,^58^ magnetic sensors,^59^ hard disk drive *etc*^60^.

**Fig. 7:**
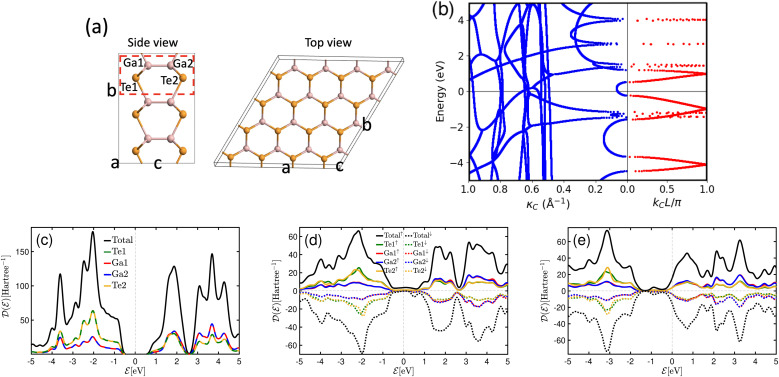
(a) Side and top views of the atomic structure of monolayer GaTe. Dashed rectangular shows its unit cell. (b) Calculated complex band structures of bulk GaTe, *L* is the layer separation perpendicular to the cleave plane. Both the real bands (right panel) and imaginary bands (left panel) are plotted. Projected density of states for (c) freestanding monolayer GaTe, and monolayer GaTe in the PtTe_2_/F4GT/GaTe/F4GT/PtTe_2_ heterostructure for (d) parallel and (e) antiparallel configurations.

In our investigation, we have introduced GaTe as a barrier between the ferromagnetic electrodes. GaTe is a member of the group-VIII metal chalcogenide family and is a semiconductor with an indirect bandgap. It crystallizes in the *P*6̄*m*2 space group,^61,62^ and its lattice parameter for a single layer is approximately 4.09 Å, closely matching that of F4GT and PtTe_2_. This close lattice match minimizes material mismatches in the heterostructure, promoting better interface quality. Fig. 7a presents side and top views of monolayer GaTe's atomic structure, consisting of four atoms in its unit cell: two Ga and two Te atoms.

Accurately evaluating the exponential decay of wave transmission necessitates a comprehensive analysis of the dispersion spectrum, considering both propagating and evanescent wave modes. In Fig. 7b, we present the computed complex band structures of bulk GaTe, showcasing the real bands on the right panel and the imaginary bands on the left. Here, *L* in the imaginary bands represents the set at 17.52 Å for semiconducting GaTe with AB stacking. The right panel of the plot illustrates the real bands, while the left part exhibits the complex bands plotted against the imaginary part, *κ*_C_. The calculated band gap of 0.78 eV for bulk GaTe aligns with findings from ref. 63. Evanescent states exhibit a characteristic decay length inversely proportional to the imaginary wave vector *κ*_C_. Thus, our primary focus is on states where *κ*_C_ remains small within the gap. Notably, the presence of states with increasing *κ*_C_ indicates a progressive enhancement of the damping or broadening parameter in the complex band structure, signifying the temporal decay of electronic states.

The total and atomic projected density of states for freestanding monolayer GaTe is displayed in Fig. 7c, revealing a semiconductor phase with a band gap of approximately 1.05 eV. Notably, the projected density of states for atoms of the same type within the unit cell exhibits similar behavior. To investigate changes in the electronic structure of monolayer GaTe when integrated into the PtTe_2_/F4GT/GaTe/F4GT/PtTe_2_ heterostructure, we computed the spin-polarized projected density of states for both parallel and antiparallel configurations. These results are presented in panels (d) and (e) of Fig. 7, respectively. Our findings indicate that the incorporation of a GaTe monolayer in the device heterostructure leads to the observation of non-zero states in the DOS of GaTe at the Fermi level in both configurations. These states are known as metal-induced gap states (MIGS) and are responsible for the tunneling process. Moreover, the behavior of the density of states for atoms of the same type within the unit cell is altered due to the influence of the electrodes. MIGS arise from the interaction between the GaTe monolayer and the adjacent metallic electrodes. Their formation can be described by two primary mechanisms. The first mechanism involves charge transfer at the interface, where the wave functions of the metal penetrate into the semiconductor's band gap in the energy range where the metal's conduction band overlaps with the semiconductor gap. This interaction generates a continuum of metal-induced gap states derived from the virtual gap states of the semiconductor's complex band structure. Additionally, MIGS can be associated with intrinsic interface states on the semiconductor side, which may pin the Fermi level. These intrinsic states create localized energy levels within the band gap, further enhancing the presence of MIGS at the interface.^64^ Furthermore, in panel d, it is observed that in the parallel configuration, the monolayer GaTe becomes polarized as a result of its interaction with the ferromagnetic electrodes. In contrast, no such polarization is observed in the antiparallel configuration, as depicted in panel e. This lack of polarization in the antiparallel configuration arises from the opposing spin orientations in the left and right electrodes, which effectively cancel out their individual polarization effects on the barrier. Moreover, the magnetic moment calculations for the device with parallel configuration confirm a slight polarization of GaTe when it is positioned between two F4GT layers. The magnetic moments of Te and Ga are found to be 0.002 *μ*_B_ and 0.004 *μ*_B_, respectively. These results suggest that GaTe experiences a subtle magnetic influence in the heterostructure, due to its interaction with the adjacent F4GT layers.

**Fig. 8:**
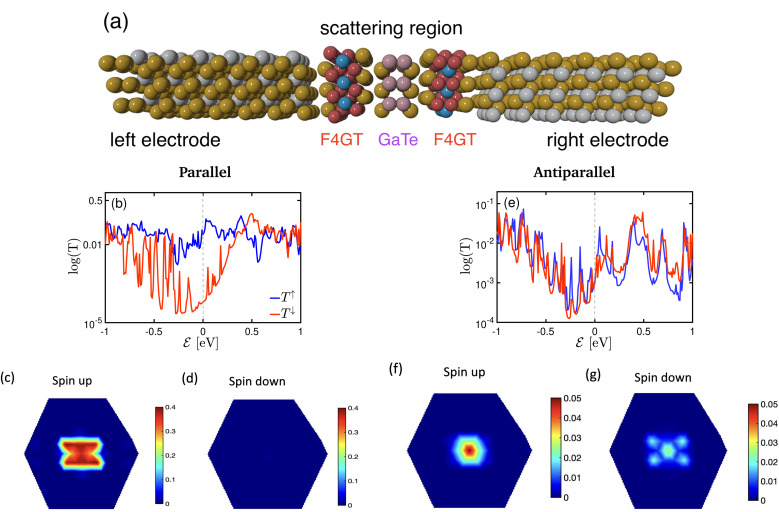
(a)Two-probe model used for NEGF calculations, depicting a Fe_4_GeTe_2_/monolayer GaTe/Fe_4_GeTe_2_ magnetic tunnel junction sandwiched between two PtTe_2_ electrodes. Transmission characteristics of the system in the (b) parallel and (e) antiparallel state under zero bias voltage on a logarithmic scale. *k*_‖_-resolved transmission probability at the Fermi energy for (c and f) spin-up and (d and g) spin-down for (c and d) parallel and (f and g) antiparallel state at zero bias voltage.

A schematic view of a single layer of GaTe as a spacer between two layers of F4GT, creating a heterostructure of PtTe_2_/F4GT/monolayer GaTe/F4GT/PtTe_2_ is shown in Fig. 8a. The distance between the GaTe layer and left (right) F4GT electrodes was obtained as 3.2 (3.08) Å which is less than the distance between GaTe layers in AB stacking form (3.81 Å). Fig. 8 indicates the transmission probability for the parallel (P) and antiparallel (AP) spin states in panels (b–d) and (e–g), respectively. Upon introducing GaTe as a spacer between F4GT layers, a decrease in the transmission probability is observed, as shown in panel 8b in comparison to Fig. 6b. In Fig. 8b, a very high spin polarization is observed in the transmission spectrum for the P state at the Fermi level, indicating a preference for one spin orientation over the other. Notably, near the Fermi level, perfect spin filtering occurs, where the transmission is non-zero only for the spin-up channel. This demonstrates that the system acts as an efficient spin filter, allowing only spin-up electrons to pass through the constriction. This nearly perfect spin polarization of transmission can be attributed to the specific electronic and magnetic properties of the F4GT layers in MTJ. In the parallel configuration, the aligned magnetic moments of the F4GT layers create a coherent magnetic environment that favors the transmission of spin-up electrons, minimizing scattering and enhancing spin-dependent transport. The interface between F4GT and PtTe_2_ acts as an efficient spin filter, where spin-up electrons experience less resistance, contributing more to the transmission while spin-down electrons are suppressed. High-quality interfaces between the layers ensure minimal scattering and defects, which is critical for maintaining high spin polarization. Additionally, the alignment of specific orbitals, such as the d-orbitals of Fe and the p-orbitals of Te, supports efficient spin-up electron transport in the parallel configuration.

The *k*_‖_-resolved transmission probability at the Fermi energy for the spin-up and spin-down channels, presented in panels (c) and (d) of Fig. 8, shows that the transmission channels for spin-up electrons are significantly higher than those for spin-down electrons. There is a relatively large transmission probability only around the *Γ* point (*k*_‖_ = 0) in the 2D Brillouin zone for the spin-up channel (Fig. 8c), indicating efficient spin-filtering due to aligned magnetic moments. Electron tunneling is highly sensitive to momentum conservation laws. The *Γ* point, being a high-symmetry point in the Brillouin zone, has lower in-plane momentum (*k*_‖_), which makes it easier for these electrons to tunnel through the barrier with minimal scattering, resulting in higher transmission probabilities near this point. This high transmission probability is consistent with typical behavior in ferromagnetic MTJs, where majority spins encounter less resistance. For spin-down electrons (Fig. 8d), the transmission probability is significantly lower, demonstrating the spin-filtering effect.

In the AP configuration (Fig. 8e), the system exhibits reduced spin polarization and less efficient spin filtering behavior near the Fermi level. However, the transmission for up and down-spin electrons is not the same in the AP configuration. This discrepancy is due to slight asymmetries in the atomic structure at the interfaces of F4GT and GaTe after relaxation, as shown in Fig. S4.† These asymmetries can lead to differences in the magnetic moments of Fe atoms in the F4GT layers and the spin-dependent transmission for an antiparallel configuration of the MTJ. These structural asymmetries, along with differences in the magnetic moments of Fe atoms, contribute to the observed variations in spin-dependent transport properties. The transmission probability through the device is less sensitive to the electron spin orientation, resulting in a more balanced transmission for both spin-up and spin-down electrons. As can be seen in Fig. 8f and g, in the antiparallel configuration, the *k*_‖_-resolved transmission probabilities for both spin-up and spin-down electrons are notably reduced compared to the parallel configuration. The reduced transmission in *k*_‖_ space and the absence of transmission channels in regions away from the *Γ* point indicate that the transmission is confined to a specific momentum range for both spin orientations. This confinement and reduced transmission efficiency are due to the anti-aligned magnetic moments at the interfaces, leading to increased scattering and less efficient electron transport. Consequently, the transport properties of the system in the AP configuration suggest a less pronounced spin-dependent behavior compared to the P configuration. This observation is consistent with the reported values for P and AP transmission of F3GT/h-BN/F3GT and F3GT/graphene/F3GT heterostructures.^45^ Furthermore, we calculated the resistance–area (RA) product from the transmission using the formula 
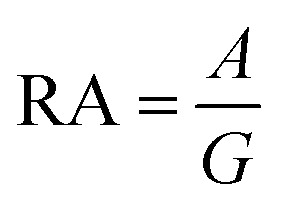
, where 
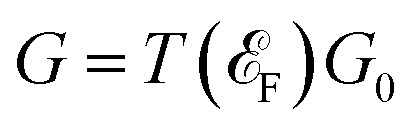
 and 
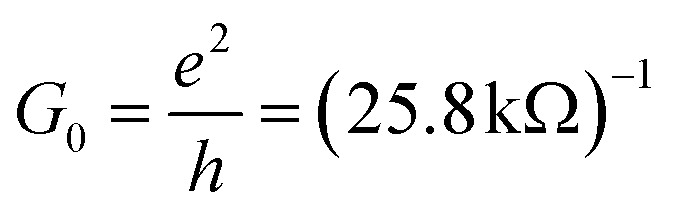
 is the spin-conductance quantum. Here, *A* is the unit cell area of our setup, calculated as 
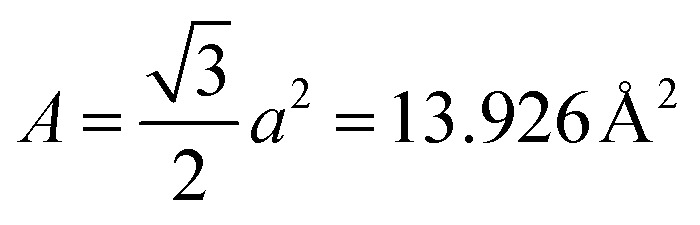
, with *a* = 4.01 Å being the in-plane lattice constant of the device. Then, the resistance-area product is:4
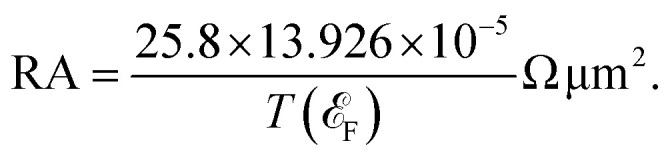


For the PtTe_2_/F4GT/GaTe/F4GT/PtTe_2_ MTJ, the resistance–area values obtained are 0.137 Ω μm^2^ and 1.618 Ω μm^2^ for parallel and antiparallel configurations, respectively. It is evident that when the MTJ is in the parallel state, it exhibits lower resistance, resulting in higher transmission values than in the antiparallel state.

**Fig. 9:**
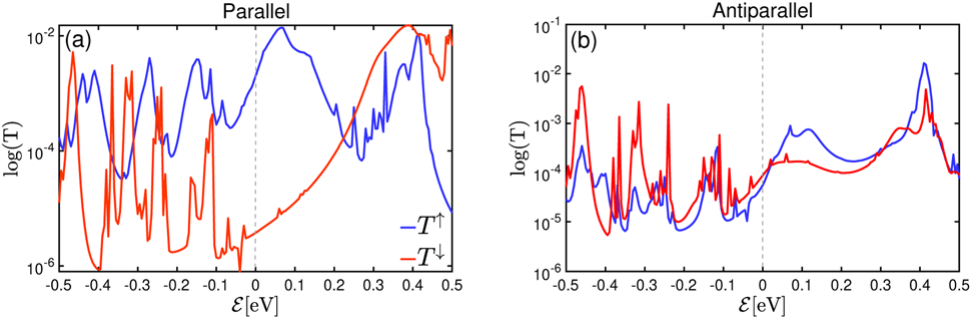
Transmission characteristics of the Fe_4_GeTe_2_/bilayer GaTe/Fe_4_GeTe_2_ in the (b) parallel and (b) antiparallel state under zero bias voltage on a logarithmic scale.

Moreover, we focused on the implementation of a bilayer of GaTe as the barrier layer. Transmission coefficient of the Fe_4_GeTe_2_/bilayer GaTe/Fe_4_GeTe_2_ in the (b) parallel and (b) antiparallel state under zero bias voltage is shown in Fig. 9. The total transmission coefficients at the Fermi level for the Fe_4_GeTe_2_/bilayer GaTe/Fe_4_GeTe_2_ system were determined to be 0.002 and 1.4316 × 10^−4^ in the parallel and antiparallel magnetic states under zero bias voltage, respectively. Notably, these values are lower than the total transmission coefficients observed when utilizing a monolayer of GaTe as the barrier layer, where they were found to be 0.0250 and 0.0021 for the parallel and antiparallel magnetic states under zero bias voltage, respectively. This outcome highlights the introduction of a bilayer of GaTe as a more effective hindrance to electron transport in the MTJ. Specifically, the bilayer GaTe barrier demonstrates significantly reduced transmittance in both parallel and antiparallel magnetic configurations compared to its monolayer counterpart.

The total charge current of the device is calculated as the sum of two components: *I* = *I*↑ + *I*↓ (eqn (3)). By measuring the currents at different voltages, the TMR ratio can be determined using the formula:^65^5
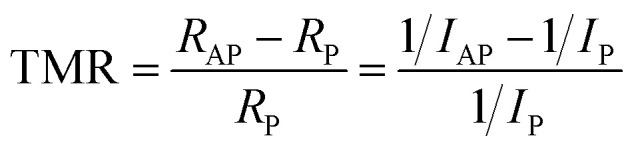
where *R*_P_ and *R*_AP_ denote the resistances in the P and AP, respectively. Similarly, *I*_P_(*I*_AP_) represents the total charge currents in the *P* (AP) magnetization configurations.

**Fig. 10:**
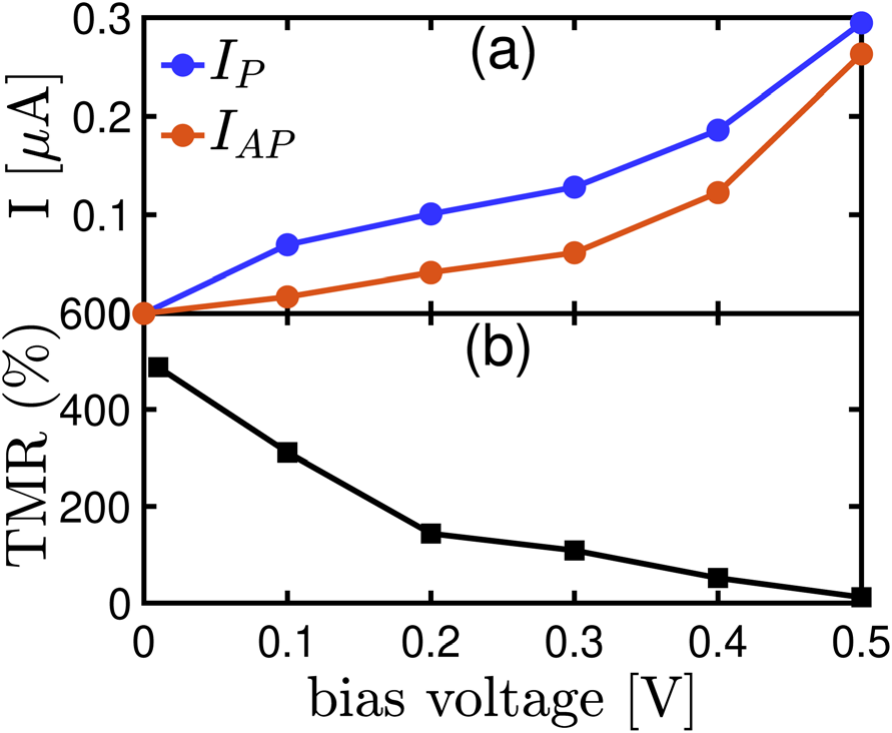
(a) Total current, a summation of both spin-up and spin-down currents, of Fe_4_GeTe_2_/monolayer GaTe/Fe_4_GeTe_2_ magnetic tunnel junction sandwiched between two PtTe_2_ electrodes at the parallel and antiparallel state as a function of bias voltage. (b) Variation of the TMR with bias voltage for the same MTJ.

Fig. 10a illustrates the *I*–*V* curve for the Fe_4_GeTe_2_/monolayer GaTe/Fe_4_GeTe_2_ magnetic tunnel junction sandwiched between two PtTe_2_ electrodes in both parallel and antiparallel configurations. The current in the P state is higher than the current in the AP state under the given bias voltage, indicating more efficient electron transport when the magnetic moments of the Fe_4_GeTe_2_ layers are aligned parallel. This suggests that electrons with parallel spins have a higher probability of transmitting through the device, leading to more efficient transport of charge carriers. This behavior is consistent with the spin-filtering effect observed in the P state (Fig. 8b), where the majority spins experience less resistance and are favored in the transport process. On the other hand, in the AP state, the transmission of both spin-up and spin-down (Fig. 8e–g) electrons is more restricted due to the anti-alignment of the magnetic moments. As a result, the total current in the AP configuration is reduced compared to the P configuration.

Tunnel magnetoresistance is a key parameter used to quantify the difference in resistance between the P and AP configurations of an MTJ. A higher TMR indicates a more efficient spin-filtering effect and a larger difference in current between the two spin states, while a lower TMR suggests a reduced difference in current. In Fig. 10b, TMR is plotted for PtTe_2_/Fe_4_GeTe_2_/monolayer GaTe/Fe_4_GeTe_2_/PtTe_2_ MTJ within a bias range starting from 0.01 V, as it requires a finite bias voltage for accurate representation. The system exhibits a high TMR of 487% at low bias, surpassing the reported TMR value of 89% for PtTe_2_/F4GT/α-In_2_Se_3_/F3GT/PtTe_2_ heterostructures.^2^ However, as the bias voltage increases, the TMR gradually decreases, reaching 12% at 0.5 V. This decline suggests that the efficiency of the spin-filtering effect diminishes under higher voltage conditions. The higher voltage leads to stronger carrier injection in the device, which can modify the spin-dependent transport properties and result in the observed reduction in TMR. Additionally, the investigation into spin filtering and the potential utilization of other members within the FGT family has produced notable findings. For example, an experiment involving a spin valve device integrated a vertical F3GT/h-BN/F3GT magnetic MTJ with an electrolyte gate, resulting in a MR ratio of 36% for the intrinsic MTJ. Electrolyte gating further enhanced the TMR ratio of the F3GT/h-BN/F3GT heterostructure from 26% to 65%^33^ which is less than the observed value of TMR for F4Gt-based MTJ, especially in low bias voltages. Also, a TMR value of 141% has been documented in van der Waals magnetic heterostructures comprising F3GT and FePSe_3_ at 5 K.^12^ The ability to modulate magnetic fields and magnetoresistance switches presents a promising avenue for controlling the magnetization configuration of the MTJ. In the context of F3GT/Cr_2_Ge_2_Te_6_/F3GT van der Waals junctions, a transition from negative-to-positive magnetoresistance was observed with an increasing bias voltage.^32^ This transition, attributed to the changing spin polarizations, was supported by calculated spin-dependent density of states under bias conditions.

## 4 Conclusion

In conclusion, we have investigated the spin-dependent transport properties of Fe_4_GeTe_2_/GaTe/Fe_4_GeTe_2_ vdW heterostructures sandwiched between PtTe_2_ electrodes using first-principles calculations and non-equilibrium Green's function method. We analyzed the electronic DOS of F4GT in both freestanding and device configurations, revealing its ferromagnetic metallic nature and sensitivity to local environments. Through our study, we have demonstrated the formation of spin valves with well-defined spin filtering behavior. Transmission eigenstates of a monolayer F4GT sandwiched between PtTe_2_ reveal interference patterns influenced by relative phases and localization differing in spin-up and spin-down channels. The transport characteristics of a double-layer F4GT with a ferromagnetic configuration, placed between two PtTe_2_ electrodes, are found to display remarkable spin polarization of 97%. This indicates a strong tendency for one spin orientation to dominate the transport process. The transport properties of F4GT-based MTJs by introducing monolayer GaTe as a spacer between F4GT layers show a remarkable value of 487% of TMR at low bias surpassing the existing values reported for similar systems in literature. TMR decreases with increasing bias voltage, indicating the modification of spin-dependent transport properties under carrier injection. These findings open up new opportunities for the design and optimization of spintronic devices based on FGT and related heterostructures, advancing the field of spintronics and offering the potential for future technological advancements.

## Conflicts of interest

There are no conflicts to declare.

## Supplementary Material

NA-006-D4NA00639A-s001

## Data Availability

All the data presented in this paper are available from the authors.
