# Supplementary material for: Efficient spin filtering through Fe4GeTe2-based van der Waals heterostructures
Source: Nanoscale Adv. 2024 Oct 9;6(24):6278–89. doi: 10.1039/d4na00639a (PMC11485126; doi:10.1039/d4na00639a)
Supplement: NA-006-D4NA00639A-s001 [file NA-006-D4NA00639A-s001.pdf]

Supplementary Information (SI) for Nanoscale Advances.
This journal is © The Royal Society of Chemistry 2024

Efficient spin filtering through Fe_4GeTe_2-based van der Waals heterostructures

Masoumeh Davoudiniya[1] and Biplab Sanyal[1,*]

[1] *Department of Physics and Astronomy,*
Uppsala University, Box-516, 75120 Uppsala, Sweden

(Dated: August 1, 2024)

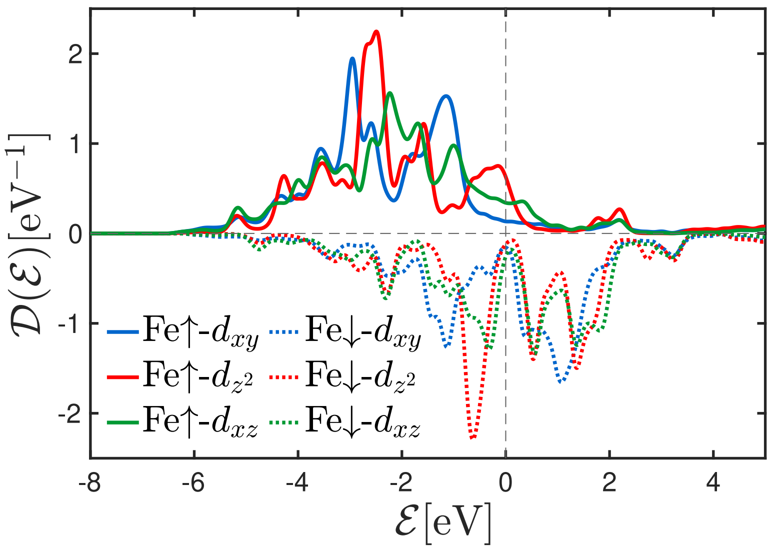

FIG. S1. Orbital-decomposed d-band density of states for the 3d orbitals of Fe atoms in Fe_4GeTe_2 (F4GT). The Fermi level is set at 0 eV. The solid lines represent the DOS of the spin-up channel, while the dotted lines represent the DOS of the spin-down channel. The DOS for the d_{xz} and $d_{x^2-y^2}$ orbitals are identical. Similarly, the d_{yz} and d_{xz} orbitals also exhibit identical DOS.

I. DOS OF MONOLAYER F4GT

Figure S1 illustrates the spin-polarized density of states of Fe 3d orbitals in Fe_4GeTe_2, highlighting significant spin-splitting and contributions to the states at the Fermi level. The DOS for the d_{xz} and $d_{x^2-y^2}$ orbitals are identical. Similarly, the d_{yz} and d_{xz} orbitals also exhibit identical DOS. From the DOS plot, it is evident that the Fe 3d orbitals contribute significantly to the states at the Fermi level. Specifically, the d_{xz} and d_{z^2} orbitals show pronounced spin-split peaks near the Fermi level, with the spin-up states contributing more prominently than the spin-down states. The d_{xy} orbitals also contribute, though less significantly. This spin-splitting indicates strong magnetic interactions within F4GT, impacting its spin-dependent transport properties.

* Biplab.Sanyal@physics.uu.se

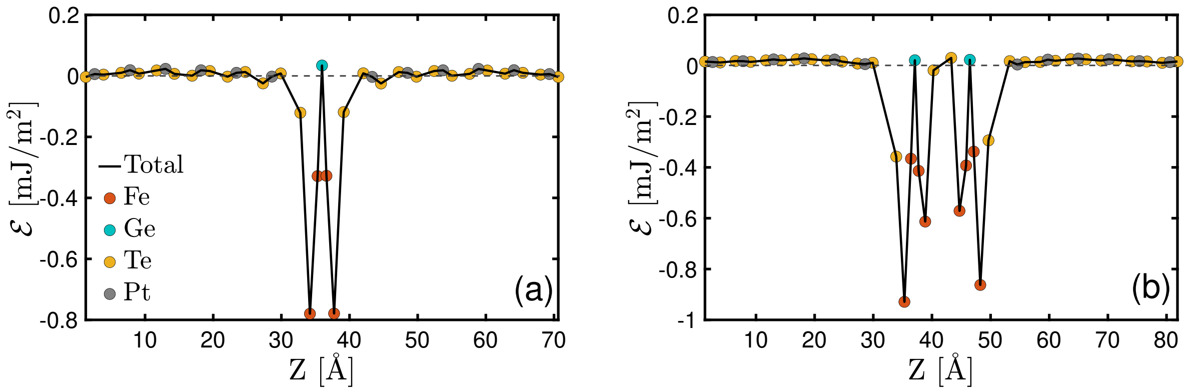

FIG. S2. Atom projected in-plane magnetocrystalline anisotropy energy plots for (a) mono- and (b) bi-layer F4GT sandwiched between two $PtTe_2$ electrodes.

II. STABILITY ANALYSIS OF HETEROJUNCTIONS

We have investigated the stability of the heterojunctions, focusing on the energy stability of different interfaces within the materials. We calculated the total energy of freestanding monolayer structures, including F4GT ($E_{tot} = -2192.69$ eV), PtTe2 ($Etot = -2192.69$ eV), and GaTe ($E_{tot} = -2192.69$ eV). To assess the energy differences between the interfaces, we considered $PtTe_2$/F4GT and F4GT/GaTe with total energies equal to -31182.29 eV and -31790.3 eV, respectively.

The energy difference (ΔE) can be written as follows:

ΔE = Total Energy[F4GT/$PtTe_2$] - {Energy[F4GT] + Energy[$PtTe_2$]}

ΔE = Total Energy[F4GT/GaTe] - {Energy[F4GT] + Energy[GaTe]}

The results reveal that ΔE for the F4GT and $PtTe_2$ interface is -0.77 eV, while ΔE for the F4GT and GaTe interface is -0.21 eV. These negative values indicate that the heterojunctions exhibit a lower total energy than the sum of the energies of the individual layers. This suggests a favorable condition for the stability of the heterojunctions compared to the separate layers, confirming the robustness of our findings.

III. MAGNETIC ANISOTROPY ENERGY

In spintronic devices, the behavior of charge transport is highly dependent on how the magnetic moments in the materials are aligned. To investigate this relationship, we calcu-

TABLE I. Comparison of the atom projected MAE (in unit of mJ/m^2) for mono- and bi-layer freestanding F4GT with the device configuration, where F4GT is sandwiched between two layers of $PtTe_2$. Values in parentheses for bilayer cases refer to the MAE of corresponding atoms in the second layer.

	**Monolayer**		**Bilayer**	
	freestanding	device	freestanding	device
Fe1	-1.08	-0.78	-0.67 (-0.9)	-0.62 (-0.857)
Fe2	-0.44	-0.33	-0.40 (-0.39)	-0.41 (-0.34)
Fe3	-0.44	-0.33	-0.39 (-0.41)	-0.37 (-0.39)
Fe4	-1.12	-0.78	-0.89 (-0.68)	-0.93 (-0.57)
Te1	-0.35	-0.12	-0.24 (-0.45)	-0.02 (-0.29)
Te2	-0.38	-0.12	-0.45 (-0.25)	-0.36 (-0.03)
Ge	-0.03	-0.03	0.005 (0.004)	0.02 (0.02)
**Total**	**-3.84**	**-2.17**	**-6.09**	**-4.55**

lated the MAE of (a) single- and (b) bi-layer F4GT connected to $PtTe_2$ electrodes in Fig. S2. The MAE values were calculated using the force theorem [1]. The MAE quantifies the energy difference between two spin orientations aligned along the easy axis (the preferred direction) and the hard axis (the unfavorable direction) of the material, which are described by the spherical angles θ and ϕ, $MAE = \mathcal{E}(\theta_1, \phi_1) - \mathcal{E}(\theta_0, \phi_0)]$. The obtained results demonstrate a substantial in-plane MAE at the scattering part for the device configurations. This finding aligns with the reported MAE value of freestanding F4GT, which also indicates an in-plane easy axis in various calculations [2, 3]. The weak interaction at the interface between F4GT and the electrodes does not change the direction of the MAE.

For a comprehensive investigation of the electrode's impact on the MAE of F4GT, we calculated the atomic MAE values for both mono- and bi-layer freestanding F4GT, as well as the device configuration with F4GT placed between two layers of $PtTe_2$ (see Tab. I). All values are given in units of mJ/m^2, and for the bi-layer cases, the values in parentheses indicate the MAE of corresponding atoms in the second layer. We observed that the direction of MAE remains consistent across all cases, indicating that the preferred magnetic orientations in F4GT persist regardless of the presence of electrodes. However, the absolute

value of MAE decreases when F4GT is connected to the electrodes. This decrease signals the influence of the weak interaction on the stability and alignment of the magnetic moments in F4GT when placed between the electrodes.

A reduction in the MAE of F4GT in the device configuration compared to its freestanding form suggests that the magnetic moments in the material become less stable and more susceptible to changes in external conditions. The lower MAE implies that the magnetic moments in F4GT require less energy to switch their orientation. At the interfaces between F4GT and $PtTe_2$, there can be d-p electronic hybridization, as shown in the LDOS in Figure 5. This hybridization can disrupt the spin-orbit coupling that contributes to MAE. Moreover, freestanding monolayers and few-layer structures are highly sensitive to surface and interface effects. The reduction in dimensionality enhances spin-orbit coupling effects, leading to more pronounced anisotropy due to the increased confinement of electronic states and lower symmetry compared to bulk materials. Monolayers exhibit a higher surface-to-volume ratio, exposing all atoms to the surface. This exposure significantly alters the electronic structure, resulting in higher MAE. In bulk materials and device configurations, these effects are averaged out over the larger volume, resulting in lower anisotropy. This behavior is consistent with reports on the MAE of other magnetic materials, such as Gd_2 [4].

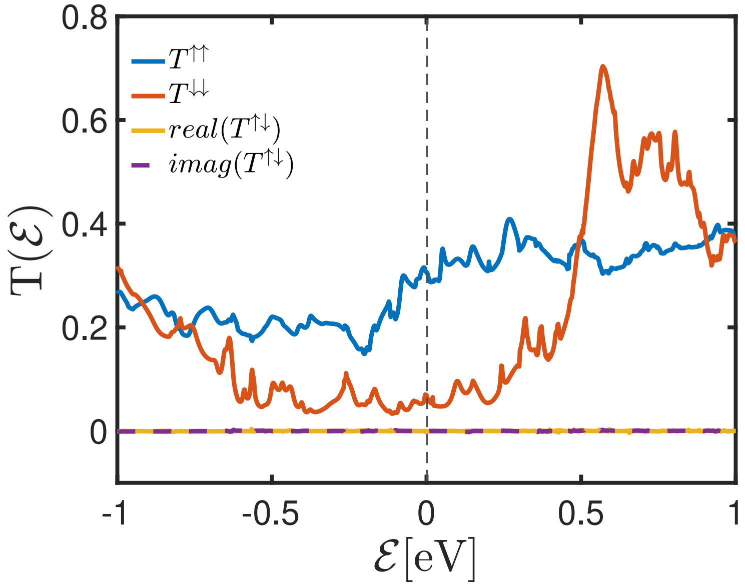

FIG. S3. The transmission spectrum of the monolayer F4GT placed between two $PtTe_2$ electrode system under zero bias voltage for noncollinear configurations.

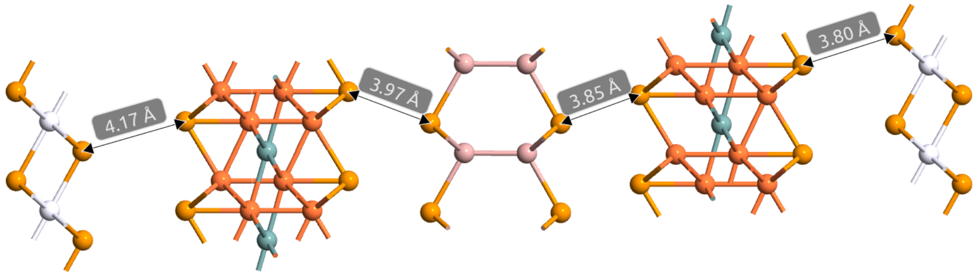

FIG. S4. Atomic structure of the Fe_4GeTe_2/GaTe/Fe_4GeTe_2 heterostructure sandwiched between $PtTe_2$ electrodes. The distances between adjacent layers are labeled: 4.17 Å between the left $PtTe_2$ electrode and the Fe_4GeTe_2 layer, 3.97 Å between the left Fe_4GeTe_2 layer and GaTe, 3.85 Å between GaTe and the Fe_4GeTe_2 layer in the right part, and 3.80 Å between the Fe_4GeTe_2 layer and the $PtTe_2$ in the right electrode. These measurements highlight the structural asymmetries present at the interfaces, which can lead to differences in the spin-dependent transmission properties.

IV. NON-COLLINEAR TRANSPORT

Fig. S3 demonstrates the zero-bias transmission coefficient of the device consisting of a monolayer F4GT placed between two $PtTe_2$ electrodes, considering spin-orbit coupling. The results show that the influence of non-collinear magnetic configurations on the transport properties of the device is minimal. Consequently, we only consider the collinear magnetic configuration in the quantum transport calculations. This approach simplifies the analysis and focuses on the primary magnetic configuration that significantly impacts the transport properties.

V. MAGNETIC TUNNEL JUNCTION

As shown in the figure S4, after relaxation, there are slight asymmetries in the atomic structure at the interfaces of Fe_4GeTe_2 and GaTe, which can lead to differences in the spin-dependent transmission for an antiparallel configuration of MTJ. This asymmetry is also found in the parallel state, where the magnetic moments of Fe atoms differ between the left and right F4GT layers. Specifically, the magnetic moments are:

- Left layer: Fe1 = 2.734 μ_B, Fe2 = 1.733 μ_B, Fe3 = 1.743 μ_B, Fe4 = 2.744 μ_B

- Right layer: Fe1 = 2.732 μ_B, Fe2 = 1.725 μ_B, Fe3 = 1.736 μ_B, Fe4 = 2.727 μ_B

These variations in magnetic moments and structural asymmetries result in differences in the transmission for up-spin and down-spin electrons, even in the anti-parallel configuration. Consequently, the transmission for up and down-spin electrons is not identical for a MTJ with anti-parallel configuration as shown in Fig. 8(e).

---

[1] G. H. O. Daalderop, P. J. Kelly, and M. F. H. Schuurmans, First-principles calculation of the magnetocrystalline anisotropy energy of iron, cobalt, and nickel, Phys. Rev. B **41**, 11919 (1990).

[2] S.-R. Kim, I. K. Park, J.-G. Yoo, J. Seo, J.-G. Kim, J.-H. Park, J. S. Kim, K. Kim, G. Lee, and K.-T. Ko, Role of orbital bond and local magnetism in fe3gete2 and fe4gete2: Implication for ultrathin nano devices, ACS Applied Nano Materials **5**, 10341 (2022).

[3] X. Yang, X. Zhou, W. Feng, and Y. Yao, Strong magneto-optical effect and anomalous transport in the two-dimensional van der waals magnets $\mathrm{fe}_n\mathrm{gete}_2$ (n = 3, 4, 5), Phys. Rev. B **104**, 104427 (2021).

[4] Y. Wang, M. Yang, Z. Cui, H. Zeng, X. Zhang, J. Shi, T. Cao, and X. Fan, Monolayer and bilayer lanthanide compound gd2c with large magnetic anisotropy energy and high curie temperature, Journal of Materials Science **58**, 268 (2023).
